# Countywide burden, pathology, and genetics of lethal hypertrophic cardiomyopathy: from the POST SCD study

**DOI:** 10.1093/europace/euaf088

**Published:** 2025-04-18

**Authors:** Leila Haghighat, Andrew Connolly, Francesca Nesta Delling, Theodore Pravinchandra Abraham, Ellen Moffatt, Zian H Tseng

**Affiliations:** Division of Cardiology, Department of Medicine, University of California-San Francisco, San Francisco, 500 Parnassus Avenue, Box 1354, CA 94143-1354, USA; Department of Pathology and Laboratory Medicine, University of California-San Francisco, San Francisco, CA, USA; Division of Cardiology, Department of Medicine, University of California-San Francisco, San Francisco, 500 Parnassus Avenue, Box 1354, CA 94143-1354, USA; Division of Cardiology, Department of Medicine, University of California-San Francisco, San Francisco, 500 Parnassus Avenue, Box 1354, CA 94143-1354, USA; Department of Pathology and Laboratory Medicine, University of California-San Francisco, San Francisco, CA, USA; Office of the Chief Medical Examiner, San Francisco, CA, USA; Division of Cardiology, Department of Medicine, University of California-San Francisco, San Francisco, 500 Parnassus Avenue, Box 1354, CA 94143-1354, USA

**Keywords:** Sudden death, hypertrophic cardiomyopathy, arrhythmic death, cardiac genetics

## Abstract

**Aims:**

Incidence of sudden cardiac death (SCD) is 1%/year in cohorts with hypertrophic cardiomyopathy (HCM), but this estimate presumes arrhythmic cause and misses occult cases dying before diagnosis.

**Methods and results:**

POST SCD (POstmortem Systematic InvesTigation of Sudden Cardiac Death) is a prospective cohort study using autopsy, clinical records, and toxicology to adjudicate arrhythmic or non-arrhythmic causes among presumed SCDs (pSCDs) meeting WHO criteria aged 0–90 years in San Francisco County. We included all incident cases 2/1/2011–3/1/2014 (*n* = 525) and approximately every third day 3/1/2014–9/1/2022 (*n* = 497) based on medical examiner call schedule. We identified HCM victims via three approaches: (i) pathology; (ii) echocardiogram [transthoracic echocardiogram (TTE)]; (iii) genetic criteria. Incidence calculations used county data and estimated HCM prevalence of 1:500 from studies of persons aged 23–35 years old. Of 1022 pSCDs [558 (54.6%) arrhythmic deaths] during the study period, 13 had HCM: 10 met pathology criteria; 2 via review of 203 TTEs (missed on initial report); 1 via genetic testing. Of these, 11 were arrhythmic deaths, yielding 1.3% burden of sudden death (pSCD) and 2% of arrhythmic death. Only 2 of 13 (15%) pSCDs with HCM had pre-mortem diagnosis. Incidence for persons with HCM 18–35 years old was 0.2% pSCDs/year and 0.1% SADs/year. pSCDs with HCM had a higher proportion of arrhythmic cause [11/13 (85%) vs. 547/1009 (54%), *P* = 0.03] than those without. pSCD burden due to HCM decreased with age (*P* = 0.003), highest among victims <35 years old, for whom HCM accounted for 7.1% of pSCD and 9.4% of arrhythmic death. Genetic testing of 317 consented pSCDs yielded pathogenic or likely pathogenic variants in 40% (2/5) and identified one additional case without clinical phenotype.

**Conclusion:**

In this 11-year countywide post-mortem study, HCM meeting pathologic, clinical, or genetic criteria was associated with autopsy-confirmed arrhythmic cause of sudden death, accounting for 2% of SADs up to age 90, highest in cases <35 years old. Since 85% of cases were undiagnosed before pSCD, the true burden of HCM-related sudden death may be substantially underestimated.

## Graphical Abstract

**Graphical Abstract:**
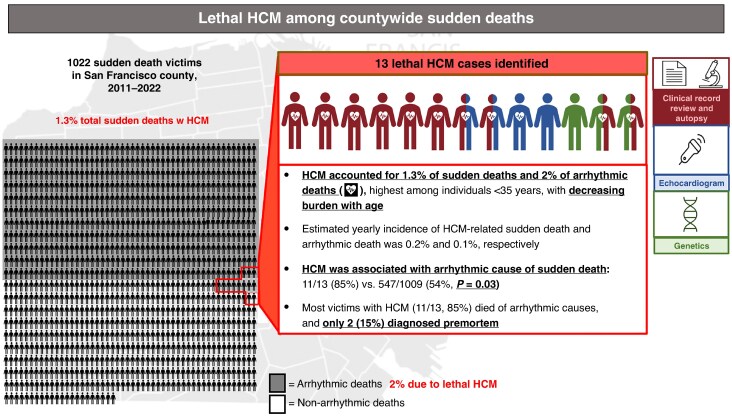

**What’s New**

- In this 11-year countywide study using comprehensive criteria with echocardiography, pathology, and genetics, hypertrophic cardiomyopathy (HCM) accounted for 1.3% of all sudden deaths and 2% of arrhythmic deaths in the community, highest among individuals <35 years, with decreasing burden by age. Yearly incidence of HCM-related sudden death was 0.2% and HCM-related arrhythmic death was 0.1%.
- While the contribution of HCM to community sudden death is relatively modest, most victims die of arrhythmic causes potentially preventable with a defibrillator.
- The majority of sudden death victims with HCM remain undiagnosed, thus the true burden of HCM-related sudden death, traditionally based on studies of *known* disease, may be substantially underestimated.

## Introduction

Hypertrophic cardiomyopathy (HCM) is a genetic disorder of cardiomyocytes that affects up to 1:500 adults, based on echocardiographic surveillance studies of persons aged 23–35 years old.^1–3^ Mutations in at least eight sarcomere genes have been linked to HCM and have a minimal prevalence of 1:200 people.^2^ Retrospective analyses of patients with known HCM indicate that sudden cardiac death (SCD) accounts for up to 1000 cases per 100 000 person-years each year (1%) and is the most common cause of death, especially among younger individuals with the disease.^4–7^ In this population, yearly implantable cardioverter defibrillator (ICD) therapy rates are 3–5% for primary prevention and 11% for secondary prevention.^8–12^ From the pathology perspective, autopsy studies of sudden death in young individuals estimate an incidence between 0.5–39 per 100 000 person-years living with HCM and that 3.7–14% of SCDs in this demographic are attributable to HCM.^13–18^ However, these studies did not examine pre-mortem data such as echocardiograms or genetic testing to enhance detection of disease, and potentially preventable sudden arrhythmic deaths (SAD) are not distinguished from total sudden deaths.

Thus, the actual incidence of arrhythmic death among those with known and occult HCM, particularly beyond the well-studied younger demographic, remains unknown since the epidemiology of HCM is based on cases of known, clinically diagnosed HCM. Patients with HCM may die suddenly without a clinical diagnosis ever having been made, thus current estimates of prevalent HCM and incident sudden death may be based on incomplete capture of disease. Moreover, nearly all SCD studies use definitions that presume cardiac cause, such as those from the World Health Organization (WHO) and 2016 consensus society guidelines.^19,20^ These definitions rely on emergency medical services (EMS) records, death certificates, and conventional criteria,^21–23^ rather than post-mortem data to verify cause of sudden death, thus they describe *presumed* SCDs (pSCDs) as per new nomenclature.^24^

In our prospective Postmortem Systematic InvesTigation of Sudden Cardiac Death (POST SCD) Study of all pSCDs in San Francisco County, only half (55.8%) were SADs potentially rescuable with ICD. Leveraging POST SCD, we sought to evaluate the precise burden of total community sudden death attributable to HCM via comprehensive evaluation of pre-mortem studies (clinical history, echocardiograms), comprehensive post-mortem investigation, and genetic testing, and to determine the incidence of HCM-related sudden death (i.e. pSCD) and SAD.

## Methods

The University of California, San Francisco Institutional Review Board approved this study and waiver of informed consent, because all patients were deceased. This study was additionally approved by the 10 adults hospital and 3 EMS agencies that comprise San Francisco County (2022 total population: 808 437).^25^

### Study population

A full description of the methods and design of POST SCD has been previously published.^26^ Briefly, POST SCD is an ongoing prospective cohort study of consecutive outside-of-hospital cardiac arrest deaths up to 0–90 years old in San Francisco County that undergo full autopsies, including toxicology and histology. Deaths attributable to WHO-defined SCD (i.e. pSCD) complete a full post-mortem investigation to adjudicate the cause of death.

In the initial POST SCD cohort from 1 February 2011 to 1 March 2014, all 525 outside of hospital cardiac arrests (OHCAs) reported to the Office of the Medical Examiner were included. In the extended cohort, every incident case approximately every third day was included based on medical examiner (author E.M.) call schedule. The extended cohort included an additional 497 cases between 2 March 2014 and 1 September 2022. While extended cohort data could not be used in incidence estimates because it no longer captured consecutive countywide pSCDs, the use of a random selection method to mitigate selection bias (all incident cases every third day based on examiner call schedule) allowed for calculations of total burden of pSCD and arrhythmic death attributable to HCM.

Outside of hospital cardiac arrests were defined using Cardiac Arrest Registry to Enhance Survival criteria.^27^ These included deaths that: (i) were witnessed and/or active resuscitation was performed in the field or emergency department; or (ii) were unwitnessed with the victim last seen alive and symptom-free within 24 h, with no active resuscitation but a primary EMS impression of cardiac arrest. Cases were excluded if the victim survived to hospital admission (thus did not die suddenly after cardiac arrest) since these are considered resuscitated OHCAs, a condition distinct from SCD, or if the victim had a known terminal illness, end stage renal disease, or an alternative identifiable non-cardiac cause of sudden death at the scene (e.g. homicide).^28^

### Adjudication of cause of sudden death

All presumed SCDs underwent full adjudication by a multidisciplinary team that included cardiac electrophysiologists, the Assistant Medical Examiner of San Francisco County, a cardiac pathologist, and a neurologist. Pre-mortem medical records were obtained by review of electronic health records in San Francisco County and individualized requests to out-of-county institutions, if next-of-kin were aware that victims received medical care elsewhere. These medical records, along with autopsy, toxicology, and histology findings, were reviewed at adjudication.

The goals of adjudication were to determine: (i) underlying cause of death; and (ii) whether the presumed SCD was due to arrhythmic cause potentially rescuable with an ICD (hereafter SAD). Specifically, SAD was a presumed SCD for which no identifiable non-cardiac cause (e.g. pulmonary embolism, haemorrhage, and occult overdose) or cardiac non-arrhythmic cause (e.g. heart failure with pulmonary oedema, cardiac tamponade) was found on comprehensive post-mortem investigation.

### Pathological evaluation for hypertrophic cardiomyopathy

All gross heart specimens were sectioned at the apex, mid-wall, and base, and were examined for evidence of cardiovascular pathology. Any wall thickness ≥1.7 cm was considered possible HCM to account for increased concentric thickening described in rigor mortis.^29^ Kidneys and brain were carefully evaluated for signs of pathology (e.g. renal or neurovascular disease) consistent with hypertension, including in cases without documented history, to exclude hypertension as cause of LV hypertrophy. Cases with initial histologic evaluation consistent with possible amyloidosis underwent Congo red staining.

All cases underwent histological analysis of transverse sections at the septum, mid-inferior, and mid-superior left ventricular free wall. Five micrometre sections were stained with haematoxylin–eosin and Heidenhain trichrome, and independently examined by two pathologists (authors E.M. and A.C.). Hypertrophied cardiomyocytes and interstitial fibrosis have been considered sensitive markers of HCM.^30^ Specific markers of HCM include cardiomyocyte disarray and arteriolar fibroplasia.^31^ Cases met pathologic criteria if maximal wall thickness was ≥1.7 cm and either cardiomyocyte disarray, interstitial fibrosis, or hypertrophied cardiomyocytes were present.

### Echocardiographic evaluation for hypertrophic cardiomyopathy

Every transthoracic echocardiogram (TTE) performed as part of decedent’s clinical care was retrieved. All reports were reviewed to identify studies with hypertrophy (any wall thickness ≥1.5 cm or at least ‘moderate hypertrophy’ if no dimension measurements were reported), based on guideline recommendations.^32^ Images meeting these criteria were reviewed with an expert HCM echocardiographer (author T.A.). To meet echocardiographic criteria for HCM, cases had to demonstrate hypertrophy and at least one additional finding consistent with HCM: asymmetric septal hypertrophy (ASH, defined as septal-to-posterior wall thickness ratio of ≥1.3), systolic anterior motion of the mitral valve leaflet (SAM), or presence of left ventricular gradient.^33^ Other established clinical conditions known to cause hypertrophy (e.g. hypertension, severe valvular disorder, and infiltrative disease) were considered in TTE evaluation to exclude as HCM such cases of ‘explained’ hypertrophy. These were ascertained by medical record review, including consideration of past medical history and medication list. Hypertension was considered the primary cause for cases with findings of concentric hypertrophy inclusive of the apex, rather than apical sparring observed in non-apical variants of HCM.^34^

### Genetic analysis

For cases with next-of-kin consent, we performed next-generation exome sequencing of a comprehensive 245-gene panel of published and reportable monogenic cardiovascular disease genes related to arrhythmia syndromes, familial hypercholesterolaemia, HCM, dilated cardiomyopathy, arrhythmogenic right ventricular cardiomyopathy, isolated and syndromic aortopathies, thrombophilia, and rare genetic syndromes that can present with SCD (see Supplementary material online, *Tables S1* and *S2*) on DNA extracted from whole blood.^35^ Variants were classified according to the American College of Medical Genetics standards.^36^ Pathogenic, likely pathogenic, and variants of uncertain significance were reported. Cases were reviewed by the multidisciplinary team for phenotypic concordance.

### Incidence rate calculations

Data from the initial POST SCD cohort were used for calculation of incidence rates, since all incident countywide OHCAs were included in this study period. We estimated total person-years with HCM in San Francisco County as the product of published HCM prevalence of 1:500 and San Francisco County data on individuals in these age groups during the study period obtained from U.S. Census Bureau.^3,37,38^ Incidence rates per 100 000 person-years were calculated as the total number of pSCD or SAD with HCM in the initial cohort, divided by the estimated total person-years <35 years old with HCM in San Francisco County, then multiplied by 100 000. Detailed calculations are included in Supplementary Methods.

### Guideline recommendations for hypertrophic cardiomyopathy management and indications for implantable cardioverter defibrillator

To determine appropriateness of management including ICD implantation, we referenced the 2024 American Heart Association/American College of Cardiology Guideline for the Diagnosis and Treatment of Patients with Hypertrophic Cardiomyopathy.^39^

### Statistical analysis

We compared characteristics of pSCD victims with and without HCM using *χ*^2^ tests for categorical variables, and *χ*^2^ test for trend in proportions for contribution to SCD and SAD burden by age group. Stata Version 17.0 (StataCorp LP, College Park, TX, USA) was used to perform all analyses, with two-tailed *P* < 0.05 considered statistically significant.

## Results

A total of 1022 presumed SCDs were included, comprising 525 cases in the initial POST SCD cohort (2/1/11–3/1/14), and 497 victims in the extended cohort (3/2/14–9/1/22); 558 cases (54.6%) were adjudicated as SADs. Proportions of SAD among pSCDs were similar in the initial and extended cohorts (55.8% vs. 53.0%, *P* = 0.37).

**Figure 1:**
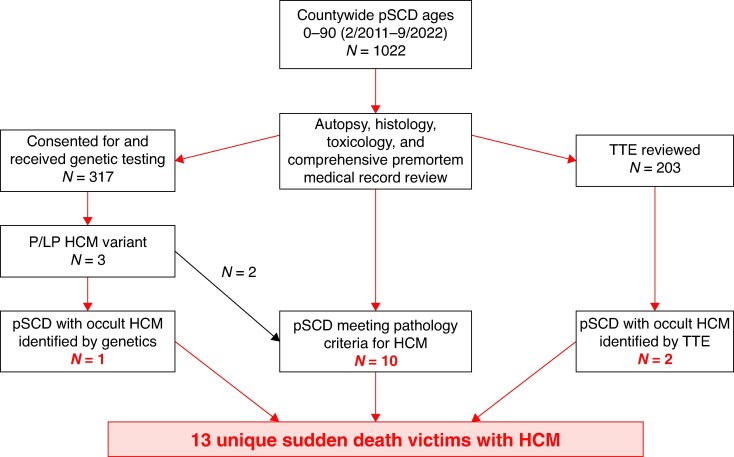
Identification of lethal HCM in the San Francisco POST SCD study 2/1/2011–9/1/2022. HCM, hypertrophic cardiomyopathy; pSCD, presumed sudden cardiac death meeting World Health Organization criteria; TTE, transthoracic echocardiogram.

**Figure 2:**
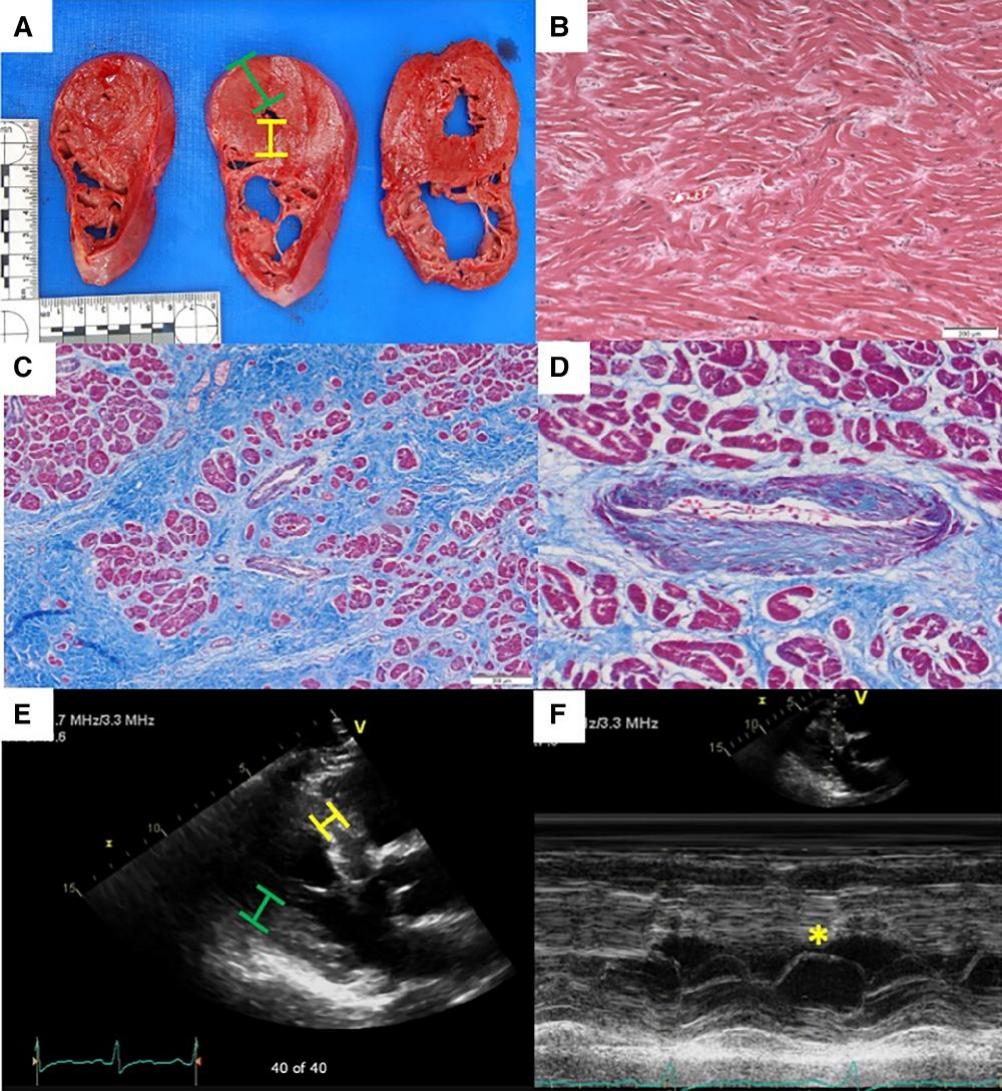
Pathologic and echocardiographic criteria for lethal HCM. (*A*) Gross pathologic specimen of a SCD victim, demonstrating left ventricular hypertrophy by autopsy criteria of wall thickness ≥1.7 cm. Green bar indicates lateral wall, and yellow bar indicates septal wall. Septal-to posterior wall thickness ratio of ≥1.3 was considered asymmetric septal hypertrophy (ASH). (*B*) Haematoxylin and eosin (H&E) stain at 10-fold magnification demonstrating cardiomyocyte disarray, a specific histologic finding for HCM. (*C*) Trichrome stain at two-fold magnification demonstrating interstitial fibrosis, a sensitive histologic finding for HCM. (*D*) Trichrome stain at 20-fold magnification demonstrating arteriolar fibroplasia, a specific histologic finding for HCM. (*E*) Parasternal long-axis images of a transthoracic echocardiogram (TTE) from an HCM SCD victim, demonstrating hypertrophy (any wall thickness ≥1.5 cm) and ASH, a specific finding for HCM. (*F*) M-mode imaging across mitral valve (MV) in a TTE of an HCM SCD victim, demonstrating systolic anterior motion (SAM) of the MV leaflet, a specific finding for HCM.

Among pSCDs, we identified 13 victims with HCM (*Figure 1*). Ten victims were adjudicated with HCM based on pathology criteria (*Figure 2*), two of which had known HCM. TTE had been performed in 203 victims of pSCD. We obtained and reviewed these reports, of which 23 had suspicion for HCM. We then reviewed the images of these 23 studies and identified four cases with TTE findings consistent with HCM, two of which did not meet pathology criteria nor carry a pre-mortem HCM diagnosis: one in a pattern of apical HCM, two reverse curve HCM, and one sigmoidal HCM. Genetic testing of 317 consented pSCDs identified pathogenic or likely pathogenic (P/LP) variants in two of the five consented cases meeting pathologic or TTE criteria, and one additional case without clinical phenotype. Thus, among all victims of presumed SCD countywide during the 11-year study period, we identified 13 total victims with HCM: 10 meeting pathologic criteria; 4 meeting clinical (TTE) criteria, 2 without pathologic features of HCM; and 3 meeting genetic criteria, 1 without pathologic or TTE features of HCM.

### Burden of sudden death and arrhythmic death attributable to hypertrophic cardiomyopathy

Hypertrophic cardiomyopathy victims thus accounted for 1.3% (13/1022) of all sudden deaths (i.e. pSCDs) during the 11-year study period. Among pSCDs with HCM, 11 of 13 (85%) had autopsy-confirmed arrhythmic cause of sudden death, including 10 attributed to HCM without other identifiable cause of sudden death and 1 due to acute myocardial infarction. HCM accounted for 11 of 558 (2%) SADs over the 11-year period. Two presumed SCDs with HCM died of non-cardiac causes of sudden death: one due to acute pulmonary embolus, one due to occult overdose.

Compared to pSCDs without HCM (*n* = 1009), those with HCM were younger (median age 52 vs. 60 years old, *P* = 0.01) and more likely to have arrhythmic cause of sudden death [11 of 13 (85%) vs. 547 of 1009 (54%), *P* = 0.03, *Table 1*].

**Table 1:** Characteristics of sudden deaths in San Francisco county with and without HCM 2/1/11–9/30/2022

	Presumed SCDs with HCM	Presumed SCDs without HCM	*P*-value
	*n* = 13	*n* = 1009	
Demographic characteristics			
Median age, years [range]	52 [16–65]	60 [0–92]	**<** **0.01**
Male (%)	11 (85)	734 (73)	0.82
White race	4 (31)	533 (53)	0.11
Medical history			
Diabetes	2 (15)	208 (21)	0.64
Hypertension	3 (23)	484 (48)	0.07
Tobacco use	5 (38)	363 (36)	0.85
CAD	2 (15)	148 (15)	0.94
CKD	0 (0)	83 (8)	0.28
AF/AFL	0 (0)	77 (8)	0.30
Arrhythmic cause of sudden death	11 (85)	547 (54)	**0.03**
Medication prescriptions			
Antiarrhythmics	0 (0)	66 (7)	0.34
Beta-blockers	1 (8)	244 (24)	0.17
Calcium-channel blockers	2 (15)	136 (13)	0.84
ACE inhibitors	0 (0)	233 (23)	0.05
ARBs	0 (0)	68 (7)	0.33
Nitrates	1 (8)	65 (6)	0.86
Diuretics	2 (15)	219 (22)	0.58

ACE, angiotensin converting enzyme; AF/AFL, atrial fibrillation/atrial flutter; ARB, angiotensin receptor blocker; CAD, coronary artery disease; CKD, chronic kidney disease; HCM, hypertrophic cardiomyopathy; SCD, sudden cardiac death.

**Figure 3:**
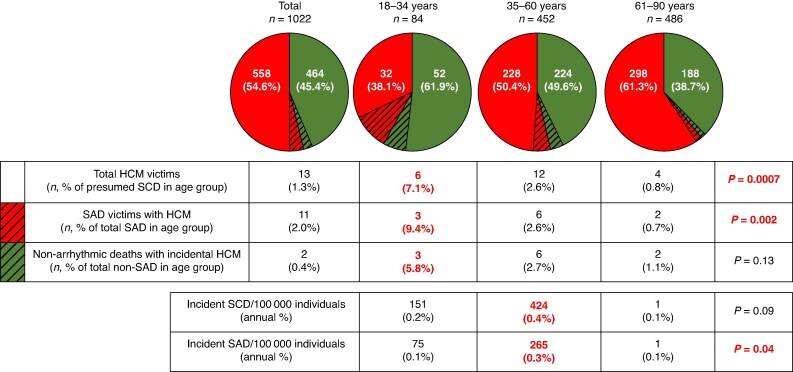
Hypertrophic cardiomyopathy contribution to sudden death by age. SAD, sudden arrhythmic death; SCD, sudden cardiac death.

Sudden death and SAD burden attributable to HCM was highest among victims <35 years old, decreasing with age (*P* = 0.002, *Figure 3*). Specifically, among victims <35 years old, HCM accounted for 7.1% of all sudden deaths and 9.4% of SADs.

### Estimated countywide incidence of sudden death and arrhythmic death among persons with hypertrophic cardiomyopathy

Estimated annual incidence of sudden death ranged from 0.1 to 0.4% across all age groups, highest among victims 35–60 years old (424 sudden deaths per 100 000 person-years living with HCM; 0.4% annually). Estimated annual incidence of SAD ranged from 0.1 to 0.3%, highest among victims 35–60 years old (265 SADs per 100 000 person-years living with HCM; 0.2% annually).

### Pathologic vs. echocardiographic criteria for hypertrophic cardiomyopathy

We compared clinical, pathologic, echocardiographic, and genetic features of the victims of sudden death with HCM (*Table 2*). Two victims met both pathology and TTE criteria, one met pathologic but not TTE criteria, two met TTE but not pathologic criteria, and eight met only pathologic criteria. Both cases who met only TTE criteria exhibited dynamic findings of HCM, including SAM, and left ventricular intra-cavitary or outflow tract gradient, that are not detectable on autopsy. A single patient who had low left ventricular ejection fraction but no other echocardiographic evidence of HCM may have represented burnt-out HCM, but lack of clinical history or pulmonary edema on autopsy consistent with congestive heart failure makes this unlikely.

**Table 2:** Comparison of diagnostic criteria for identification of lethal HCM victims

	Patient 1	Patient 2	Patient 3	Patient 4	Patient 5	Patient 6	Patient 7	Patient 8	Patient 9	Patient 10	Patient 11	Patient 12	Patient 13
Pathology criteria met	**+**	**+**	**+**	−	−	+	+	+	+	+	+	+	+
TTE criteria met	**+**	**+**	−	**+**	**+**	−	−	−	−	−	−	−	−
Genetics criteria met	−	+	**+**	−	−	−	−	−	−	−	−	−	−
Clinical features													
Age	52	42	59	60	57	54	52	18	64	16	65	20	25
History of cardiac arrest or sustained ventricular arrhythmia	No	No	No	No	No	No	No	No	No	No	No	No	No
History of syncope suspected to be arrhythmic	No	Yes	No	No	No	No	No	No	No	No	No	No	No
History of non-sustained ventricular arrhythmia	N/A	No	N/A	N/A	N/A	No	No	No	No	No	No	No	No
Family history of HCM	No	No	Yes	No	No	No	No	No	No	No	No	No	No
BMI (kg/m^2^)	31	27	41	32	37	44	22	21	26	33	31	27	38
Pathologic features													
Maximal wall thickness (cm)	2.5*	3.5*	2.0*	2.0*	3.0*	2.0*	2.0*	2.0*	1.7*	1.7*	1.7*	2.5*	2.2*
Heart mass Z-score	2.0	3.1	−0.4	2.3	2.3	1.3	3.8	1.6	−0.7	1.6	3.8	3.1	0.2
Cardiomyocyte disarray	No	Yes*	No	Not available	Not available	Yes*	No	Yes*	Yes*	Yes*	No	No	No
Hypertrophied cardiomyocyte	Yes*	No	No	Not available	Not available	No	No	Yes*	Yes*	Yes*	No	No	Yes*
Interstitial fibrosis	Yes*	Yes*	Yes*	Not available	Not available	Yes*	Yes*	Yes*	Yes*	No	Yes*	Yes*	No
Echocardiographic features													
Ejection fraction	60–65%	73%	45–50%	60%	70%	N/A	N/A	N/A	N/A	N/A	N/A	N/A	N/A
Maximal wall thickness (cm)	1.5^a^	2.0^a^	No hypertrophy noted	2	2.6^a^	N/A	N/A	N/A	N/A	N/A	N/A	N/A	N/A
Asymmetric septal hypertrophy	Yes^a^	Yes^a^	No	Yes^a^	No	N/A	N/A	N/A	N/A	N/A	N/A	N/A	N/A
Systolic anterior motion of mitral valve leaflet	No	No	No	Yes^a^	Yes^a^	N/A	N/A	N/A	N/A	N/A	N/A	N/A	N/A
Left ventricular intra-cavitary or outflow tract gradient	Yes, 13 mmHg^a^	No	No	Yes, 29 mmHg^a^	No	N/A	N/A	N/A	N/A	N/A	N/A	N/A	N/A
Left ventricular aneurysm	No	No	No	No	No	N/A	N/A	N/A	N/A	N/A	N/A	N/A	N/A
Genetic features													
P/LP mutations (variant)	N/A	*MYBPC3*	*TNNC1*	N/A	N/A	None	None	N/A	N/A	None	N/A	N/A	None
		c.2905C>T (p.Gln969)	c.175dupG										
Adjudication													
Cause of death	Arrhythmic	Arrhythmic	Arrhythmic	Pulmonary embolism	Myocardial infarction	Arrhythmic	Arrhythmic	Arrhythmic	Arrhythmic	Arrhythmic	Arrhythmic	Arrhythmic	Chemical overdose

Victims met pathology criteria if any left ventricular wall thickness was ≥1.7 cm and an additional histologic marker of HCM was identified, including cardiomyocyte disarray, interstitial fibrosis, or hypertrophied cardiomyocytes. Instances in which pathologic criteria for HCM were met are noted in the table with an asterisk (*), and instances in which echocardiographic criteria for HCM were met are noted with an ^a^. Victims met echocardiographic criteria if either septal or posterior wall thickness ≥1.5 cm, or in cases where images were not available if the report denoted at least moderate hypertrophy. In addition, to rule in for HCM by echocardiography, at least one additional echocardiographic finding consistent with HCM had to be present, including asymmetric septal hypertrophy, systolic anterior motion of the mitral valve leaflet, or presence of a left ventricular gradient. N/A refers to instances where testing was not completed, such as lack of ambulatory monitoring for evaluation of non-sustained ventricular arrhythmia, echocardiogram, or genetic testing.

BMI, body mass index; HCM, hypertrophic cardiomyopathy; TTE, transthoracic echocardiogram.

### Yield of genetic testing for hypertrophic cardiomyopathy in POST SCD

Of the 12 victims meeting pathologic or TTE criteria for HCM, the next-of-kin of 5 consented to genetic testing; 2 were found to have pathogenic or likely pathogenic (P/LP) variants associated with HCM—a yield of 40% (2/5). These included *P* variants in *MYBPC3* in a case with HCM diagnosed pre-mortem (c.2905C > T) and *TNNC1* in case with occult (i.e. undiagnosed) HCM (c.175dupG) (see Supplementary material online, *Table S3*). The next-of-kin of 312 additional victims of pSCD (without pathologic or TTE findings of HCM) consented for and underwent genetic testing. Of these, we found an additional P/LP variant in *ALPK3* (c.3526G > T) in a victim who died of occult overdose.

### Identification of potentially preventable hypertrophic cardiomyopathy sudden deaths countywide

Among the 11 victims of arrhythmic death with HCM, 2 cases (18%) had been diagnosed before death, both of whom had undergone incomplete pre-mortem risk stratification while 1 had met guideline criteria for ICD implantation but did not receive one (see Supplementary material online, *Table S4*). Thus, at the population level over 11 years, current community practice and screening had diagnosed only 18% (2/11) of potentially preventable arrhythmic deaths with HCM countywide and led to appropriate ICD implantation in only 9% (1/11).

## Discussion

In this 11-year countywide post-mortem study, we report the precise burden of sudden death and arrhythmic death attributable to HCM by comprehensive analysis including autopsy, clinical, and genetic data. Our key findings are as follows: (i) the population burden of sudden death and arrhythmic death up to 90 years old attributable to HCM were 1.3 and 2%, respectively, highest among cases 0–35 years old; (ii) HCM is associated with SAD, as arrhythmic cause of sudden death was found in 85% of cases with HCM but only half of cases without HCM; (iii) estimated incidence of HCM-related sudden death was highest among middle-aged individuals; (iv) the vast majority of cases were undiagnosed before sudden death, thus the true burden of HCM-related sudden death may be substantially underestimated; and (v) applied in the community, current guidelines would have captured <10% of potentially preventable arrhythmic deaths attributable to HCM.

This study leverages the unique, comprehensive post-mortem methodology of POST SCD to extend our prior work on the contribution of an array of high-risk conditions, recognized and occult, to the total burden of sudden death.^40–43^ Our study addresses limitations of selection bias of only younger cases in prior autopsy series evaluating the burden of HCM among sudden deaths, since all victims countywide up to the ninth decade of life were included. Thus, we were able to determine that although the highest proportion of arrhythmic deaths attributable to HCM was in cases <35 years old at nearly 10%, HCM still accounts for ∼1% of sudden deaths 61–90 years old. Moreover, we complement autopsy data with pre-mortem clinical data and genetic testing to fully detect HCM among these countywide sudden deaths, a technique that has been shown previously to increase diagnostic yield among sudden deaths.^13–18,44^ The higher cutoff of our pathologic criteria (≥1.7 cm) served to ensure a high specificity of incidence calculation, as suggested in published literature.^45^

Based on published estimates of the population prevalence of HCM and our precise determination of sudden death and arrhythmic deaths in San Francisco County, we estimate annual incidence rates of 0.25 and 0.15% respectively, among persons living with HCM countywide. Our findings are more consistent with other autopsy-based studies, which also found annual incidence rates of less than 1%, but lower than the SCD incidence rates of 0.2–0.7% based on prospective cohort studies.^4–7,13–15^ Potential reasons for the lower incidence we found include the stringent criteria we used to define SCDs in our numerator, and inflation of the numerator in other prospective cohort studies by including survivors of appropriate ICD shocks. While our study design did not include resuscitated cardiac arrest victims who subsequently died in the hospital, such cases did not die suddenly out-of-hospital and are thus not considered presumed SCDs. Therefore, our results suggest that while the widely cited incidence rates of HCM SCD based on cohort studies of *diagnosed* HCM do not account for cases with occult HCM, they inflate the incidence of HCM SCD by presuming cardiac cause of sudden death.

Notably, we report that sudden deaths with HCM were more often arrhythmic than total sudden deaths (85% vs. 58%, *P* = 0.04). The preponderance of arrhythmic deaths among all HCM-related sudden deaths observed in our study underscores that the majority of these SCDs are potentially preventable. This corroborates data from prospective HCM cohorts, which indicate that SCD is the most common cause of death in this population, followed by heart failure, stroke, and complications of heart transplantation.^4,10^

Current guidelines for ICD implantation were met in only 1 of 11 HCM-related SADs countywide. While most victims did not have a complete work-up of HCM pre-mortem thus precluding consideration for ICD, the fact that none had received one may indicate gaps in current guideline criteria that miss patients without overt structural or conduction system pathology associated with HCM, but who are nevertheless at high risk for arrhythmic death. For example, patients with a P/LP variant in the sarcomere gene *MYBPC3*, such as one case in our study who had a clinical diagnosis of HCM pre-mortem, are at particularly high risk for ventricular arrhythmias.^46^ At present, the incorporation of genetic data into individualized HCM SCD risk estimation and the need for primary prevention ICD remains controversial. Based on an association of sarcomere mutations with higher rates of adverse outcomes, 2022 European guidelines for SCD prevention recommend ICD implantation in HCM patients with intermediate 5-year risk of SCD and an established P/LP variant.^47–50^ However, 2023 European guidelines on cardiomyopathies do not incorporate genetic results, given the lack of data demonstrating their independent association with SCD risk.^51^ Instead, these guidelines rely on the validated HCM risk-SCD score for adults.^52^ American guidelines similarly do not incorporate results of genetic testing into consideration of ICD implantation.^39^

Most patients with HCM in this study (85%) were found to have occult HCM, indicating that the majority of victims of SCD who die with HCM are not diagnosed in their lifetime. Incidence rate of HCM from Olmsted County using clinical records was estimated at 2.5–6.6/100 000 person-years.^53,54^ Therefore, while a direct comparison between San Francisco and Olmsted counties cannot be made, our study suggests that clinical records-based studies of HCM may substantially underestimate true disease prevalence. While EKGs were obtained in five HCM victims in this study, all showing either left ventricular hypertrophy or diffuse T-wave inversions, only two of these patients ever received a clinical diagnosis of HCM (see Supplementary material online, *Table S5*). Furthermore, even in these cases of known HCM, work-up was incomplete. Increased clinician awareness of the indicated work-up for abnormal EKGs, especially those with suggestion of HCM, or more frequent referral to specialized centres for HCM may be warranted.

### Limitations

Our study was restricted to San Francisco County, which may limit the generalizability of our findings. In addition, our incidence calculations used current nationwide estimates of HCM prevalence which do not account for cases of occult HCM. Despite our rigorous approach to account for as many cases of HCM as possible, there may have been instances of HCM in which none of our criteria were met, including cases with only mild hypertrophy. We may have also missed additional cases with P/LP variants in sudden death victims whose family did not consent to genetic testing. While victims whose next-of-kin declined autopsy were not included in our study, only 2% of POST SCD-eligible victims were missed by our protocol during the study period. The patient who ruled in for HCM based on P/LP *ALPK3* variant was included in our calculation of incidence because of being adjudicated as having possible HCM, based on maximal autopsy wall thickness of 1.5 cm. Although his death was not clearly arrhythmic, non-arrhythmic cause of death does not rule out the possibility of his having structural changes consistent with HCM. Exclusion of this patient would have lowered our reported incidence of HCM. While we recognize that HCM guidelines do not require an additional finding consistent with HCM beyond wall thickness on echocardiography, we included this in our criteria to ensure specificity, which may have underestimated incidence calculations. Use of a random selection method in the extended cohort (all incident cases every third day based on examiner call schedule) mitigated selection bias of eligible cases in the latter cohort, as evidenced by a similar proportion of arrhythmic deaths among pSCDs and a similar burden of all sudden deaths and arrhythmic deaths attributable to HCM in the initial and extended study periods.

## Conclusions

In this post-mortem study of SCDs 0–90 years old in an entire metropolitan area over an 11-year period, HCM meeting pathologic, echocardiographic, or genetic criteria accounted for 2% of potentially preventable arrhythmic deaths, the highest relative burden in middle age. Most victims of arrhythmic death with HCM were not diagnosed pre-mortem, suggesting that the true burden of HCM-related sudden death may be substantially higher than currently recognized.

## Supplementary Material

euaf088_Supplementary_Data

## Data Availability

Due to privacy concerns, the data cannot be publicly shared but can be accessed by qualified researchers with appropriate approval.
