# Supplementary material for: Countywide burden, pathology, and genetics of lethal hypertrophic cardiomyopathy: from the POST SCD study
Source: Europace. 2025 Apr 18;27(5):euaf088. doi: 10.1093/europace/euaf088 (PMC12107038; doi:10.1093/europace/euaf088)
Supplement: euaf088_Supplementary_Data [file euaf088_supplementary_data.docx]

**Supplementary Material**

**Supplementary Methods**

**Determination of Countywide Sudden Death Incidence in Persons with HCM, Initial Cohort Study Duration 2/1/2011-3/1/2014**

Person-years at risk =

Total Person-Years in San Francisco County

= 2011+2012+2013+2014 Weighted Person-Years

- 2011 Weighted Person-Years: (11 months / 12 (months/year)) * (815650) = 747679
- 2012 Weighted Person-Years: (12 months / 12 (months/year)) * (828876) = 828876
- 2013 Weighted Person-Years: (12 months / 12 (months/year)) * (839572) = 830572
- 2014 Weighted Person-Years: (2 months / 12 (months/year)) * (850750) = 141792

= 747679 + 828876 + 830672 + 141792 person-years
= 2,549,019 person-years

Age-Adjusted Total Person-Years in San Francisco County Age Group 1

= (Percent of population <35 years old) * Total Person-Years in San Francisco County

= 0.26 * 2,549,019 person-years

= 662745 person-years

Age-Adjusted Total Person-Years in San Francisco County Age Group 2

= (Percent of population 35-60 years old) * Total Person-Years in San Francisco County

= 0.37 * 2,549,019 person-years

= 943137 person-years

Age-Adjusted Total Person-Years in San Francisco County Age Group 3

= (Percent of population 35-60 years old) * Total Person-Years in San Francisco County

= 0.24 * 2,549,019 person-years

= 611765 person-years

Total Person-Years with HCM in San Francisco County Age Group 1

= Estimated Prevalence of HCM * Total Person-Years in San Francisco County

= (1/500) * 662745 person-years

= 1326 person-years with HCM

Total Person-Years with HCM in San Francisco County Age Group 2

= Estimated Prevalence of HCM * Total Person-Years in San Francisco County

= (1/500) * 943137 person-years

= 1886 person-years with HCM

Total Person-Years with HCM in San Francisco County Age Group 3

= Estimated Prevalence of HCM * Total Person-Years in San Francisco County

= (1/500) * 611765 person-years

= 1224 person-years with HCM

Incidence of SCD with HCM in San Francisco County Age Group 1

= Total number of SCD cases in POST SCD study Age Group 1 with HCM in Initial Cohort / Total Person-Years with HCM in San Francisco County * 100,000

= 2/1326 person-years with HCM * 100,000

*= 151 SCD / 100,000 person-years with HCM (0.15% annually)*

Incidence of SAD with HCM in San Francisco County Age Group 1

= Total number of SAD cases in POST SCD study Age Group 1 with HCM in Initial Cohort / Total Person-Years with HCM in San Francisco County * 100,000

= 1/1326 person-years with HCM * 100,000

*= 75 SAD/100,000 person-years with HCM (0.08% annually)*

Incidence of SCD with HCM in San Francisco County Age Group 2

= Total number of SCD cases in POST SCD study Age Group 2 with HCM in Initial Cohort / Total Person-Years with HCM in San Francisco County * 100,000

= 8/1886 person-years with HCM * 100,000

*= 424 SCD / 100,000 person-years with HCM (0.42% annually)*

Incidence of SAD with HCM in San Francisco County Age Group 2

= Total number of SAD cases in POST SCD study Age Group 2 with HCM in Initial Cohort / Total Person-Years with HCM in San Francisco County * 100,000

= 5 / 1886 person-years with HCM * 100,000

*= 265 SAD/100,000 person-years with HCM (0.27% annually)*

Incidence of SCD with HCM in San Francisco County Age Group 3

= Total number of SCD cases in POST SCD study Age Group 3 with HCM in Initial Cohort / Total Person-Years with HCM in San Francisco County * 100,000

= 1/1224 person-years with HCM * 100,000

*= 82 SCD / 100,000 person-years with HCM (0.08% annually)*

Incidence of SAD with HCM in San Francisco County Age Group 3

= Total number of SAD cases in POST SCD study Age Group 3 with HCM in Initial Cohort / Total Person-Years with HCM in San Francisco County * 100,000

= 1/1224 person-years with HCM * 100,000

*= 82 SAD/100,000 person-years with HCM (0.08% annually)*

**Table S1. HCM-related genes included in next generation exome sequencing of lethal HCM victims whose next of kin consented for testing.**

| **HCM-related genes** | | | |
| --- | --- | --- | --- |
| *ACADVL* | *CSRP3* | *LDB3* | *NEXN* |
| *ACTC1* | *DES* | *MTO1* | *PDLIM3* |
| *ACTN2* | *ELAC2* | *MYBPC3* | *PLN* |
| *AGL* | *FHL1* | *MYH6* | *PRKAG2* |
| *ALPK3* | *FLNC* | *MYH7* | *TCAP* |
| *ANKRD1* | *GAA* | *MYL2* | *TNNC1* |
| *BAG3* | *GATA4* | *MYL3* | *TNNI3* |
| *CACNA1C* | *GLA* | *MYLK2* | *TNNT2* |
| *CALR3* | *JPH2* | *MYOM1* | *TPM1* |
| *CAV3* | *KLF10* | *MYOZ2* | *TTR* |
| *CPT2* | *LAMP2* | *MYPN* | *VCL* |

**Table S2. Non-HCM-related cardiomyopathy genes included in next generation exome sequencing of lethal HCM victims whose next of kin consented for testing.**

| **Additional genes tested** | | | | | | |
| --- | --- | --- | --- | --- | --- | --- |
| *ABCC9* | *ABCG5* | *ABCG8* | *ACTA2* | *ACVRLL1* | *ANK2* | *APOA1* |
| *APOA5* | *APOB* | *APOC2* | *APOC3* | *ATP13A3* | *BMPR2* | *CACNA2D* |
| *CACNB2* | *CALM1* | *CALM2* | *CALM3* | *CASQ2* | *CAV1* | *CSTB* |
| *CYP27A1* | *DEPDC5* | *DOLK* | *DSC2* | *DSG2* | *DSP* | *EFEMP2* |
| *EIF2AK4* | *ENG* | *EPHB4* | *EYA4* | *F2* | *F5* | *FKRP* |
| *FKTN* | *FOXE3* | *GATA5* | *GATAD1* | *GDF2* | *GJA5* | *GPIHBP1* |
| *HCN2* | *HCN4* | *JUP* | *KCNA1* | *KCND3* | *KCNE1* | *KCNE2* |
| *KCNH2* | *KCNJ2* | *KCNK3* | *KCNQ1* | *LAMA4* | *LDLR* | *LDLRAP1* |
| *LIPA* | *LMF1* | *LMNA* | *LOX* | *LPL* | *MAT2A* | *MFAP5* |
| *MPL* | *MYH11* | *MYLK* | *NEBL* | *NKX2-5* | *NOTCH1* | *PCSK9* |
| *PKP2* | *PRKG1* | *PROC* | *PROS1* | *PRRT2* | *RBM20* | *RYR2* |
| *SCARB1* | *SCN10A* | *SCN1A* | *SCN2A* | *SCN5A* | *SCN8A* | *SERPINC1* |
| *SMAD3* | *SMAD4* | *SMAD9* | *SNTA1* | *TBX20* | *TECRL* | *TGFB2* |
| *TGFB3* | *TGFBR1* | *TGFBR2* | *TMEM43* | *TNNI3K* | *TRDN* | *TTN* |
| *AKAP9* | *ACTA1* | *ANGPTL3* | *AQP1* | *CACNA1A* | *CDH2* | *CETP* |
| *CREB3L3* | *CTF1* | *CTNNA3* | *CYP7A1* | *DTNA* | *FHL2* | *GATA6* |
| *GCKR* | *GPD1L* | *HTRA1* | *ILK* | *KCNA5* | *KCNE3* | *KCNE5* |
| *KCNJ5* | *KCNJ8* | *LIPC* | *LRP6* | *LRRC10* | *MIB1* | *MYL4* |
| *MYLK3* | *MYO6* | *PITX2* | *PLEKHM2* | *PRDM16* | *RANGRF* | *SCN1B* |
| *SCN2B* | *SCN3B* | *SCN4B* | *SLC2A10* | *SMAD2* | *TMPO* | *TRPM4* |
| *ABCA1* | *ANO5* | *BGN* | *BRAF* | *CAPN3* | *COL3A1* | *DMD* |
| *DNAJC19* | *EMD* | *FBN1* | *FLNA* | *GNB5* | *GPD1* | *HNRNPDL* |
| *SCN4B* | *SLC2A10* | *SMAD2* | *TMPO* | *TRPM4* | *ABCA1* | *ANO5* |
| *BGN* | *BRAF* | *CAPN3* | *COL3A1* | *DMD* | *DNAJC19* | *EMD* |
| *FBN1* | *FLNA* | *GNB5* | *GPD1* | *HNRNPDL* | *HRAS* | *KRAS* |
| *LCAT* | *LZTR1* | *MPA2K1* | *MAP2K2* | *MED12* | *MRAS* | *MTTP* |
| *MYOT* | *NF1* | *NRAS* | *PLOD* | *PNPLA2* | *PPA2* | *PPP1CB* |
| *PTPN11* | *RAF1* | *RASA1* | *RIT1* | *RRAS* | *SAR1B* | *SDHA* |
| *SGCD* | *SHOC2* | *SLC22A5* | *SLC25A4* | *SOS1* | *SOS2* | *SPRED1* |
| *SYNE1* | *SYNE2* | *TAZ* | *TBX4* | *TBX5* | *TOR1AIP1* | *TXNRD2* |
| *A2ML1* | *ALMS1* | *CBL* | *CRYAB* | *KCNQ2* | *KCNQ3* | *KCNT1* |
| *PCDH19* | *SCN9A* | *SLC2A1* | *TMEM70* |  |  |  |

**Table S3. Details of genetic testing among consented sudden death victims with HCM.** Bold font signifies P/LP variants known to be associated with HCM. Remainder of variants are variants of unknown significance. Strength of evidence for HCM was based on review of information on ClinVar (<https://www.ncbi.nlm.nih.gov/clinvar/>) and ClinGen (<https://clinicalgenome.org/>). Abbreviations: Het = heterozygous, DCM = dilated cardiomyopathy, LVNC = left ventricular non-compaction, ARVD = arrhythmogenic right ventricular dysplasia, RCM = restrictive cardiomyopathy, CPVT = catecholaminergic polymorphic ventricular tachycardia.

| **Patient** | **Variant (type)** | **Associated Disease** | **HGVSc** | **HGVSp** | **Heterozygosity** | **Strength of Evidence for HCM** | **gnomAD Allele Frequency** |
| --- | --- | --- | --- | --- | --- | --- | --- |
| **1** | ***MYBPC3* (nonsense*)*** | **Autosomal dominant HCM, DCM, and LVNC** | **c.2905C>T** | **p.Gln969*** | **Het** | **Definitive** | **0.0%** |
| 1 | *APOB* (missense) | Autosomal dominant familial hypercholesterolemia | c.10579C>T | p.Arg3527Trp | Het | None | 0.0001% |
| 1 | *GAA* (missense) | Pompe | c.1726G>A | p.Gly576Ser | Het | None | 0.006% |
| 1 | *GAA* (missense) | Pompe | c.2065G>A | p.Glu689Lys | Het | None | 0.045% |
| **2** | ***TNNC1* (frameshift)** | **Familial HCM** | **c.175dupG** | **p.Glu59fs** | **Het** | **Definitive** | **0.0%** |
| 2 | *DSC2* (missense) | Familial ARVD | p.Glu2Lys | c.4G>A | Het | None | 0.0119% |
| 2 | *ENG* (splice region) | Hereditary hemorrhagic telangiectasia | Not reported | c.1853-8_1853-  3delGCTCCC | Het | None | 0.0094% |
| 2 | *DEPDC5* (missense) | Familial focal epilepsy | c.376G>A | p.Ala126Thr | Het | None | 0.0004% |
| 2 | *TTN* (missense) | DCM | c.69883G>A | p.Ala23295Thr | Het | Limited | 0.0032% |
| **3** | ***ALPK3* (stop gain)** | **Familial HCM** | **c.3526G>T** | **p.Glu1176*** | **Het** | **Definitive** | **0.0%** |
| 3 | *DTNA* (missense) | LVNC | c.1735C>T | p.Arg579Trp | Het | None | 0.0016% |
| 3 | *F5* (missense) | Thrombophilia due to activated protein C resistance | c.3841C>A | p.Leu1281Ile | Het | None | 0.0024% |
| 4 | *CACNA2D1* (missense) | Early-onset epileptic encephalopathy | c.1122C>G | p.Asn374Lys | Het | None | 0.0004% |
| 4 | *F2* (missense) | Congenital prothrombin deficiency | c.1054G>A | p.Glu352Lys | Het | None | 0.0004% |
| 5 | *ABCG8* (missense) | Sitosterolemia | c.786C>A | p.Asn262Lys | Het | None | 0.002% |
| 5 | *EYA4* (missense) | Dilated cardiomyopathy | p.Pro175Thr | c.523C>A | Het | None | 0.002% |
| 5 | *FBN1* (missense) | Marfan syndrome | p.Arg2239Ser | c.6717A>T | Het | None | 0.0% |
| 5 | *TTN* (missense) | DCM | p.Ile20900Thr | c.62699T>C | Het | Limited | 0.0012% |
| 6 | *HRAS* (missense) | Costello syndrome | p.Arg164Trp | c.490C>T | Het | None | 0.0012% |
| 6 | *KCNQ1* (missense) | Long QT syndrome | p.Val106Asp | c.317T>A | Het | None | 0.0% |
| 6 | *MYLK* (missense) | Familial thoracic aortic aneurysm | p.Arg982Cys | c.2944C>T | Het | None | 0.0008% |
| 6 | *RYR2* (missense) | Familial ARVD | p.Ser1770Asn | c.5309G>A | Het | Limited | 0.0% |
| 6 | *TTN* (missense and splice) | Autosomal recessive muscular dystrophy | p.Val467Ile | c.1399G>A | Het | Limited | 0.008% |

**Table S4. Premortem Work-up and Indications for ICD in Lethal HCM Victims.** CMR = cardiac magnetic resonance. EKG = electrocardiogram. HCM = hypertrophic cardiomyopathy. ICD = implantable cardioverter-defibrillator. LV = left ventricle. NSVT = non-sustained ventricular tachycardia. SCD = sudden cardiac death. VT = ventricular tachycardia.

|  | Known HCM (n=2) | Occult HCM (n=11) |
| --- | --- | --- |
| *Demographics*  Median age, yrs. (range)  Male  Non-white race  ICD | 47 (42-52)  1 (50)  2 (100)  0 (0) 0 (0) | 54 (18-65)  10 (91)  7 (64)  0 (0) 0 (0) |
| *Premortem work-up for HCM*  12-lead EKG  Ambulatory rhythm monitoring  Transthoracic echocardiogram  Stress testing  CMR  Genetic testing | 2 (100)  1 (50)  2 (100)  0 (0)  0 (0)  0 (0) | 3 (27)  3 (27)  3 (27)  1 (9)  0 (0)  0 (0) |
| *Indications for ICD*  Prior cardiac arrest/sustained VT/NSVT  Prior syncope  Family history of SCD/HCM  Massive LVH  LV apical aneurysm  LV systolic dysfunction | 0 (0) 0 (0)  1 (50)  0 (0)  0 (0)  0 (0)  0 (0) | 0 (0) 0 (0)  0 (0)  0 (0)  0 (0)  0 (0)  1 (9) |
| *Cause of Death*  Arrhythmic | 2 (100) | 9 (82) |

**Table S5. Diagnostic Study Results and Autopsy Characteristics in Sudden Death Victims with Known and Occult HCM.** EKG = electrocardiogram. LVEF = left ventricular ejection fraction. LVH = left ventricular hypertrophy. LVOT = left ventricular outflow tract.

|  | Known HCM (n=2) | Occult HCM (n=11) |
| --- | --- | --- |
| *EKG characteristics*  Antemortem 12-lead EKG available  LVH  Deep symmetric inverted T-waves | 2 (100)  2 (100)  1 (50) | 3 (27)  3 (27)  1 (9) |
| *Echocardiographic characteristics*  Antemortem TTE available  LVEF, % (median, range)  Hypertrophy  Septal wall thickness (cm) (median, range)  Posterior wall thickness (cm) (median, range)  Systolic anterior motion  Asymmetric septal hypertrophy  Elevated LVOT gradient | 2 (100)  68 (62.5-73)  2 (100)  1.8 (1.5-2.0)  1.1 (0.9-1.2)  0 (0)  2 (100)  0 (0) | 2 (18)  3 (47.5-73)  2 (18)  1.9 (1.1-2.6)  2.0 (1.9-2.0)  2 (18)  1 (9)  1 (9) |
